# Inflammatory polyps occur more frequently in inflammatory bowel disease than other colitis patients

**DOI:** 10.1186/s12876-020-01279-y

**Published:** 2020-06-05

**Authors:** Hassan Ashktorab, Hassan Brim, Sally Hassan, Mehdi Nouraie, Agazi Gebreselassie, Adeyinka O. Laiyemo, Angesom Kibreab, Farshad Aduli, Giovanni Latella, Steven R. Brant, Zaki Sherif, Aida Habtezion

**Affiliations:** 1 Department of Medicine, Department of Pathology and Cancer Center, Howard University Collerge of Medicine, 2041 Georgia Avenue, N.W., Washington, D.C, 20060 USA; 2 Department of Medicine, University of Pittsburgh, Pittsburgh, Pennsylvania USA; 3 Gastroenterology division, L,Aquila University, L’Aquila, Italy; 4 Division of Gastroenterology and Hepatology, Department of Medicine, Rutgers Robert Wood Johnson Medical School, and and Department of Genetics and The Human Genetics Institute of New Jersey, Rutgers University, New Brunswick, New Jersey USA; 5 Harvey M. and Lyn P. Meyerhoff Inflammatory Bowel Disease Center, Division of Gastroenterology, Johns Hopkins University School of Medicine, Baltimore, Maryland USA; 6 Gastroenterology division, Stanford University, School of Medicine, Palo Alto, California USA

**Keywords:** IBD, Colitis, African Americans, Inflammatory polyps, Adenoma

## Abstract

**Background:**

Colitis is generally considered a risk factor for colon neoplasia. However, not all types of colitis seem to have equal neoplastic transformation potential.

**Aim:**

To determine the prevalence of colorectal polyps in a predominantly African American population with inflammatory bowel disease (IBD) and Non-IBD/Non-Infectious Colitis (NIC).

**Methods:**

We retrospectively evaluated medical records of 1060 patients previously identified with colitis at Howard University Hospital, based on ICD-10 code. Among these, 485 patients were included in the study: 70 IBD and 415 NIC based on a thorough review of colonoscopy, pathology and clinical reports. Logistic regression analysis was applied to estimate the risk of polyps in patients with IBD compared to those with NIC after adjusting for age and sex. A subgroup analysis within the IBD group was performed.

**Results:**

Of the 485 patients, 415 were NIC and 70 were IBD. Seventy-three percent of the NIC patients and 81% of the IBD patients were African Americans. Forty six percent of IBD and 41% of NIC cases were male. IBD patients were younger than NIC patients (median age of 38 years vs. 50, *P* < 0.001). The prevalence of all types of polyps was 15.7 and 8.2% in the IBD and NIC groups, respectively (*P* = 0.045). Among patients with polyps, the prevalence of inflammatory polyps was higher in the IBD group (55%) compared to the NIC group (12%). After adjusting for age, sex and race, odds ratio of inflammatory polyps in IBD patients was 6.0 (*P* = 0.016). Adenoma prevalence was 4.3% (3/70) in IBD patients and 3.9% (16/415) in the NIC patients (*p* = 0.75). The anatomic distribution of lesions and colitis shows that polyps occur predominantly in the colitis field regardless of colitis type. More polyps were present in the ulcerative colitis patients when compared to Crohn’s disease patients (27% vs. 5%, *P* < 0.001) within the IBD group.

**Conclusion:**

Our study shows that inflammatory polyps are more common in IBD patients when compared to NIC patients. Most polyps were in the same location as the colitis.

## Background

Ulcerative Colitis (UC) and Crohn’s disease (CD) are the two major subtypes of Inflammatory Bowel Diseases (IBD). When patients have symptoms, signs and ileo-colonoscopic findings consistent with idiopathic IBD but cannot be classified clearly as UC or CD, they may be characterized as indeterminate colitis or IBD-Undifferentiated (IBDU) [1]. There are however many other types of colitis that are not consistent with CD, UC or IBDU. These have been referred to as non-IBD/non-infectious colitis (NIC) [2, 3].

The inflammation in UC is usually limited to the mucosal layer. It always involves the rectum, where the highest activity of inflammation is usually found, and in the majority of patients inflammation may extend more proximally in a continuous manner [4]. UC symptoms are typically characterized by chronic bloody diarrhea, tenesmus and abdominal pain.

In CD, the inflammation is transmural, affecting all layers of the gut wall and might involve any part of the gastrointestinal (GI) tract from mouth to anus. CD intestinal lesions show a discontinuous pattern most frequently affecting the terminal ileum and the colon.

About 1.4 million patients in the United States suffer from IBD. In most populations, the incidence of UC is slightly higher than CD. However, it has been observed that in African Americans CD may be more frequent than UC. A recent meta-analysis by Shi et al. reported significant variations in the demographic distribution, familial predisposition, phenotype, and outcomes of IBD between Caucasians, African Americans, Hispanics, and Asians [5]. However, in the study African Americans only made up 1% of the overall study population. Thus, there is a need for studies to understand the specific reason behind this variation in these understudied populations.

Patients with UC, as well as CD, have higher risk of dysplasia, that can be classified either as low grade dysplasia (LGD) or high grade dysplasia (HGD) [6]. African Americans are poorly represented in IBD studies despite the fact that, the population is known to be at a high risk of colonic neoplasia. NIC is a group of non-IBD colitis for which no infectious agent had been identified. Indeed apart from infectious colitis that are triggered by pathogens such as *E. coli*, *Campylobacter* and *C. difficile*, several other types of colitis are known to occur in the absence of such infectious agents [2, 3, 7–9]. These include ischemic colitis, drug-induced colitis, and radiation colitis [2]. Whether NIC represents a risk factor for the development of colorectal neoplasia is uncertain [10–13].

In IBD, colorectal neoplasia is thought to generally develop from flat low-grade or high dysplasia, or dysplasia-associated lesions or masses in the colon, now referred to as polypoid dysplasia [14]. This neoplastic transformation is triggered by multiple factors that induce several genetic and epigenetic changes in the colonocytes in a way that favors cells’ proliferation and reduce apoptosis. Dietary factors may be primary agents in such development [15–17]. In addition, the human colon contains the greatest number of bacteria, with 100-fold more genes than the human host. These bacteria, their toxins and their interaction with diet lead to the production of carcinogenic compounds that might affect the colon mucosa homeostasis [15–17]. African Americans are also known to have a high incidence of Colorectal cancer (CRC) that is more aggressive than in other populations [18]. Diet, gut microbiota and other factors trigger inflammatory reactions that might facilitate or accompany oncogenic transformation [19, 20]. Indeed, the term Colitis-Associated-Cancers (CAC) has been coined primarily to describe cancers that develop as an extension of persistent chronic inflammatory events usually IBD.

In this study, we investigated the association between IBD/NIC and polyps’ development and location in the predominantly African American population at our institution.

## Methods

### Patients

A retrospective epidemiologic investigation was conducted to identify the risk of polyp development in adult IBD patients compared with NIC patients at Howard University Hospital from 2004 to 2016. The chart review for this study was approved by our Institutional Review Board (IRB-06-MED-39).

We retrospectively evaluated the medical records of 1060 patients previously identified as colitis patients, based on ICD-9-CM and ICD-10-CM diagnostic codes. Among these, 485 patients were included in the study: 70 IBD and 415 NIC after a thorough review of their colonoscopy, pathology and clinical reports. IBD cases were identified based on diagnostic codes as follows (Table 1): 556 or K51 for UC (*n* = 33) and 555 or K50 for CD (*n* = 37). All these IBD patients meet the IBD diagnostic criteria including clinical, endoscopic and pathological findings. Among these 70 IBD patients, 81% were African Americans (AAs). NIC cases were identified based on the following ICD-9-CM or ICD-10-CM codes: 558, K52 or K55.9.

**Table 1:** ICD-9, − 10 codes associated with IBD and NIC

Type of colitis	Number of cases	ICD-9; − 10 code
Non-infectious	415	558; K52, K55.9
UC	33	556; K51
CD	37	555; K50

Amongst the NIC cases (*n* = 415), AAs represented 73% of the total group. NIC diagnosis was made by exclusion of IBD cases and known infectious colitis types (e.g. *E. coli* 0175:H7, *Campylobacter, Clostridium difficile, Cryptosporidium*). Ischemic colitis, radiation colitis, drug-induced colitis, allergic colitis, and all other non-infectious colitis types were included in the NIC group [2]. IBDU was not included in the NIC group. Colitis lesions and colonic polyps (inflammatory and non-inflammatory polyps) locations were recorded for all IBD and NIC cases. We also compared polyps in IBD vs Non-IBD/Non-Infectious Colitis with Caucasians [21–23].

### Statistical analysis

We used a table of frequency and median (interquartile range) to analyze the distribution of categorical variables and age. We tested these distributions between two groups of patients (IBD and NIC) using Fisher’s exact or chi-squared test (whichever appropriate for categorical variables) and Student’s t-test with unequal variance (for age). The association between IBD and polyps was assessed by applying the penalized Firth logistic regression model. This model is efficient in events with low frequency [24]. In each model, we used age, sex and race as potential confounders from those variables considered plausible confounders. Subgroup analyses based on race and anatomic locations of colonic lesions (colitis and polyps) were applied to data. We reported 95% confidence intervals (CI) and *P* value for all odds ratios. All models were assessed for outliers. *P* values < 0.05 were considered statistically significant and all analyses were performed in STATA 14.0 (StataCorp, College Station, TX).

## Results

### Clinical characteristics by colitis diagnosis

For the period of 2004–2016, a total of 415 Non-infectious Colitis (NIC) and 70 IBD cases involving the colon, including 33 UC patients were diagnosed at our hospital. The two groups had similar sex distribution. The median age (IQR) of IBD patients is 38 years while for NIC it is 50 years (*p* < 0.001). AAs were highly represented in both groups: 81% in the NIC group and 73% in the IBD group. The prevalence of polyps in IBD and NIC groups was 15.7% and 8.2%, respectively (*P* = 0.045, Table 2). Among all subjects (*n* = 485), and after adjusting for age, sex, and race, odds ratio of IBD for inflammatory polyps was 2.0 (95%CI = 0.9–4.2, *P* = 0.08).

**Table 2:** Demographic and clinical characteristics by colitis type

	NIC (*n* = 415)	IBD (*n* = 70)	*P* value
Age (years), Median (IQR)*	50 (39–56)	38 (28–48)	< 0.001
Male sex, n (%)	248 (60)	38 (54)	0.39
African American, n (%)	302 (73)	57 (81)	< 0.001
Hispanic, n (%)	72 (17)	1(1)	
White, n (%)	15 (4)	10 (14)	
Other, n (%)	26 (6)	2 (3)	
All types of Polyps, n (%)	34 (8.2)	11 (15.7)	0.045

*IQR Interquartile range

Among patients with polyps (*n* = 45), the frequency of the inflammatory polyps was higher in the IBD group (55%) compared to the NIC group (12%, *P* = 0.007, Table 3). After adjusting for age, sex and race, odds ratio of IBD for inflammatory polyps was 6.0 (95%CI = 1.4–25.7, *P* = 0.016). Among the patients with IBD, 45% of the polyps occurred on the left side of the colon. Another 45% of the polyps occurred on both the right and left colon while only 9% were located in the right colon (1% is missing location) (Table 4).

**Table 3:** Prevalence of inflammatory and adenomatous polyps in IBD and NIC groups

	IBD *N* = 11	NIC *N* = 34	*p* value
Inflammatory polyps, n(%)	6 (55)	4 (12)	0.007
Adenoma, n (%)	3 (27)	16 (7)	0.31
Hyperplastic, n (%)	2 (23)	14 (41)	0.30

*IBD* inflammatory bowel disease; *NIC* non-infectious colitis

**Table 4:** Comparison of polyps’ prevalence in IBD and NIC patients in relation to colitis site

Colon side	IBD	NIC
Left colon, n (%)	5 (45.5)	25 (74)
Right colon, n (%)	1 (9)	0 (0)
Bilateral, n (%)	5 (45.5)	9 (26)

*Abbreviations: IBD inflammatory bowel disease; NIC non-infectious colitis*

*2/5 both right and left in IBD group were in transverse colon.*

Adenoma prevalence was 4.3% (3/70) in overall IBD patients and 3.9% (16/415) in the NIC patients group (*p* = 0.75). Within patients with polyps, these frequencies were 27% (3/11) and 47% (16/34) in IBD and NIC patients, respectively. Most adenomas occurred in the ascending colon in both groups (58%), followed by the cecum (26%) and the transverse colon (11%).

A sub-analysis within the IBD group revealed that more polyps were present in the UC group when compared to the CD group (27% vs. 5%, *p* = 0.0001; Table 5).

**Table 5:** Prevalence of polyps in UC, CD and NIC patients

Presence of polyps	UC	CD	NIC
Yes	9 (27)	2 (5)	34 (8)
No	24 (73)	35 (95)	381 (92)

### Comparison of prevalence of polyps in IBD between African Americans and Caucasians

There are no available studies comparing the prevalence of polyps in IBD patients between patients of different racial or ethnic groups. A study conducted by Lee et al. is among the largest studies in the Caucasian population involving 1484 patients [22]. The prevalence of tubular adenoma was 4.3%, similar to our study. There were 22 (1.4%) of patients with serrated adenomas which were not present in our study. An earlier study by Dixon et al. found the prevalence of tubular adenomas to be 2.8% [21]. The number of IBD patients in that study was 106. A longitudinal study conducted by Lacucci et al. involved a total of 87 patients with IBD who were followed for 14 years [25]. This study reported higher rates of all type of polyps, tubular adenomas, and serrated adenomas in IBD patients at 59, 12.6 and 11%, respectively (Table 6).

**Table 6:** Comparison of prevalence of polyps in IBD patients in studies with predominantly African American versus Caucasian patients

Population	Prevalence of all types of polyps n (%)	Prevalence of tubular adenoma n (%)	Prevalence of serrated adenomas n (%)	References
African American n = 70	11 (15)	3 (4.3)	–	Present study ^a^
Caucasian *n* = 1484	83 (5.6)	64 (4.3)	22 (1.4)	Lee et al. (2017) ^b^
Caucasian *n* = 106	–	3 (2.8)	–	Dixon et al. (2006) ^c^
Caucasian *n* = 87	51 (59)	11 (12.6)	10 (11)	Lacucci et al. (2014)^d^

^a^Comparison between IBD and NIC; ^b^Comparison between adenomas/dysplasia and SSA/Ps only ^c^Comparison between IBD and controls; ^d^A longitudinal study

## Discussion

IBD is a chronic relapsing inflammatory disease that is distinguishable from other idiopathic inflammations affecting the gut [26]. IBD involving the colon can predispose to low and high grade dysplasia and ultimately lead to colon cancer [14]. However, there are few studies addressing this issue in AAs, a population at high risk of CRC that is witnessing a spike in IBD cases [27].

Studies show that IBD is more common in whites than other American ethnic minorities [28]. A systematic review done by Afzali et al., showed that the incidence and prevalence of IBD among minorities is increasing. Hispanics and Asians tend to get pancolonic UC [29]. There is often a significant limitation of access to treatment among minorities which predisposes them to poorer outcomes [5] A recent study that evaluated disparities of outcome among hospitalized patients with IBD, revealed that Hispanics have higher mortality when compared to non-Hispanic Whites [30]. AAs have a higher chance of recurrence of CD after surgery [31]and even AAs children have more severe disease when compared to white children [32].

The risk of CRC begins to increase 8 or 10 years after UC diagnosis with risk factors of younger age at diagnosis, longer duration, greater anatomical extent of colonic involvement, degree of inflammation, family history of CRC, and presence of primary sclerosing cholangitis [33]. Here, we analyzed patients with IBD for the presence or absence of colonic polyps. Using the ICD-9 and ICD-10 codes for colitis and a thorough review of clinical, pathological and colonoscopy reports, we identified 70 IBD and 415 NIC cases to evaluate for their correlation with colon polyp development. The two groups of patients showed similar sex distribution. AAs were highly represented in both groups: 73% in the NIC group and 81% in the IBD group. Our institution caters mainly to minority populations with a predominance of AAs. No gender difference between the two groups was noticed. There was a significant age difference between the two groups, IBD patients being younger.

CRC can develop either from an adenoma or from dysplasia developing from extended and long-lasting colitis. Another important factor of colorectal carcinogenesis is the age of the patients. Unlike the adenoma to carcinoma sequence seen in sporadic CRC patients, IBD patients likely develop carcinoma from an inflammation background [34–36]. Indeed, the prevalence of inflammatory polyps was statistically higher in IBD patients when compared to NIC patients (55% vs. 12%, *p* = 0.007).

Most inflammatory polyps do not have malignant potential. After adjusting for age, sex and race, odds ratios of IBD for inflammatory polyps was 6.0 (*p* = 0.016) among patients with polyps. The higher rate of inflammatory polyps in IBD patients might also be the normal consequence of the chronic nature of these types of colitis as opposed to NIC. In IBD, chronic active long-lasting inflammation is a risk factor for the development of CRC [37].

NIC patients seem to have a lower risk. The non-statistical difference but still observed trend (p=0.08) in the greater rate of adenomatous polyps in IBD vs. NIC patients may be due to the low number of patients included in the study, the different duration of colitis, the different ages of patients, or the different immune and molecular mechanisms of inflammation in these two groups of colitis.

The IBD population in our study was at least 12 years younger than the NIC population (38 vs. 50 years) and much younger than the recommended colorectal cancer screening age guideline of 50 years. In general, it is known that it takes at least 8–10 years for dysplasia to start to appear in IBD patients greater than that expected and guidelines recommend that patients start to have surveillance colonoscopies after this time period. The only exceptions are patients with primary sclerosing cholangitis (PSC) where annual CRC screening is to being at IBD diagnosis and there is some evidence that the risk may be earlier in patients with onset of IBD greater than 50 years [38, 39]. In patients with IBD who were followed for more than 30 years, the CRC incidence was as high as 20% and 8% for UC and CD, respectively [26-28]. Our IBD sub-group analysis revealed that more polyps were present in the UC group when compared to CD (27% vs. 5%, *p* = 0.0001) which correlates with the higher vigilance and shorter follow-ups more often employed for UC than CD patients.

The extent, severity and duration of each colitis event may determine neoplastic transformation potential. Indeed, Abu Rashed et al. reported two cases where giant obstructive polyps were displayed in a 20 year old UC patient within 1 year of diagnosis and in a 71 year old asymptomatic CD colitis case as a result of the long exposure time and duration of colitis [40].

The limitations of this study include that it is retrospective, and it lacks some clinical data in IBD patients such as duration of disease and drugs taken during the course of the disease. The age of onset of IBD in our patients was not known. Prospective studies involving both IBD and NIC with a longer time of follow up, possibly for decades are needed to clarify the role of chronic active inflammation, as well as inflammatory and non-inflammatory polyps, in the development of CRC.

Even though we did not have a large number of Caucasians in our study population, our literature comparison of the prevalence of polyps in Caucasian with IBD versus AAs was comparable in one study [22], less when compared to Dixon et al. [21] and more compared to Lacucci et al. [25]. The study by Lee et al. involved 1484 patient with IBD. There were no control groups for comparison as the purpose of the study was to determine the prevalence and character of polyps. The prevalence of adenomatous polyps was 4.3% as compared to serrated polyps which was 1.4% (*p* < 0.001). The study design by Dixon et al. was similar to our study, although it only focused on distal adenomas found during flexible sigmoidoscopy and colonoscopy. The prevalence of distal polyps was 2.8% (*p* = 0.03). The lower prevalence of adenomas is likely due to the fact that only left sided adenomas were evaluated. The study by Lacucci et al. was a cohort study which followed IBD patients for 14 years. There was no control arm in this study. The higher prevalence of polyps in this study is likely attributed to the longer period of follow up of patients.

## Conclusion

Our study showed that inflammatory polyps are more common in patients with IBD when compared to patients with other types of colitis. There were also more polyps detected in UC than CD patients. These polyps tend to develop in the same area as the colitis. The prevalence of adenomatous polyps (mostly polypoid masses with adenomatous features), although by frequency greater in IBD vs. NIC, was not statistically different. However, the numbers were limited and our study suggests that it may be valuable to examine a larger sample size to address this important question. Most studies report that inflammation plays a role in colorectal carcinogenesis, and this may include prevalence / incidence of adenomas. In IBD patients, neoplastic development occurs through a pathway where chronic inflammation seems to be the primary trigger and consistent player/stressor in the path to cancer. IBD patients with long duration of active disease need to be monitored through short interval surveillance colonoscopy as recommended in the guidelines. It is unclear if AAs need to be examined at shorter intervals. This should be a focus of future studies given their increased risk of CRC in this population.

## Data Availability

All data generated or analyzed during this study are included in this published article.

## Abbreviations

IBD: Inflammatory bowel diseases
NIC: Non-IBD/Non-Infectious colitis
UC: ulcerative colitis
CD: Crohn’s disease
GI: Gastrointestinal
LGD: Low grade dysplasia
HGD: High grade dysplasia
CAC: Colitis-associated-cancers
